# A bumper crop of SNPs in soybean through high‐density genotyping‐by‐sequencing (HD‐GBS)

**DOI:** 10.1111/pbi.13551

**Published:** 2021-02-16

**Authors:** Davoud Torkamaneh, Jérôme Laroche, Brian Boyle, David L. Hyten, François Belzile

**Affiliations:** ^1^ Département de Phytologie Université Laval Québec City QC Canada; ^2^ Institut de Biologie Intégrative et des Systèmes (IBIS) Université Laval Québec City QC Canada; ^3^ Department of Plant Agriculture University of Guelph Guelph ON Canada; ^4^ Department of Agronomy and Horticulture University of Nebraska‐Lincoln Lincoln NE USA

**Keywords:** soybean, high‐density genotyping, genotyping‐by‐sequencing, whole‐genome sequencing, imputation

Genome‐wide association studies (GWAS) have revolutionized the investigation of complex traits over the past decade and have unveiled numerous useful genotype–phenotype associations. To be comprehensive, GWAS can require identifying and genotyping hundreds of thousands to millions of genome‐wide genetic markers in large panels of accessions (Gupta *et al*., 2019). Similarly, many advances in crop genomics are closely tied to technological developments in next‐generation sequencing (NGS). In general, NGS‐based genotyping methods are classified into three categories, namely whole‐genome re‐sequencing (WGRS), SNP arrays and reduced‐representation sequencing (RRS; e.g. genotyping‐by‐sequencing (GBS)). While SNP arrays (e.g. SoySNP50K) and GBS are popular genotyping methods in many crop species, they often provide an insufficient number of markers for fine mapping and high‐resolution GWAS studies (Patil *et al*., 2016), especially when highly diverse sets of accessions need to be characterized. In contrast, WRGS can generate high‐density genome‐wide genotyping data but, when performed on a large scale (thousands of samples), it can prove quite costly.

To achieve a higher density of marker coverage than GBS or arrays, but at a lower cost than WGRS, Happ *et al*. (2019) used skim sequencing, WGRS performed at a decreased depth of coverage (0.1× to 1×), combined with imputation of missing data to capture a large number (up to ~1.3M) SNPs. Alternatively, Boudhrioua *et al*. (2020) relied on a low‐cost GBS scan followed by imputation of over 4M SNPs from a reference panel of related elite soybean lines to achieve dense genome coverage at low cost. In both cases, imputation of missing genotypes or untyped variants was required to produce complete data sets. Within the elite germplasm of many major crops, linkage disequilibrium (LD) extends over sufficiently long distances and there are enough resequenced elite lines to allow for accurate imputation in many cases. As we seek to explore the broader genetic resources, however, LD extends over shorter distances, and more markers are needed to extensively capture haplotype diversity and maintain high accuracy in imputation.

The aim of this study was to develop and establish an improved GBS approach (high‐density GBS or HD‐GBS) to significantly increase the density of detectable markers, using soybean as a test case. As it is a complexity‐reduction method, GBS relies on sequencing the optimal number of fragments falling within a specific size range. Our objective in designing HD‐GBS was to select the best combination of restriction enzymes in view of generating ~1 million fragments of 100–800 bp. To guide us in this process, we used the DepthFinder tool (Torkamaneh *et al*., 2019) and tested (*in silico*) seven different enzymes individually or in combination (*Ape*KI, *Ape*KI/*Mse*I, *Mse*I, *Msp*I/*Mse*I, *Msp*I, *Nla*III, *Bfa*I). Four of these (*Ape*KI, *Ape*KI/*Mse*I, *Msp*I/*Mse*I and *Msp*I/*Msp*I) generated ≤0.5M fragments and two (*Mse*I and *Nla*III) were predicted to generate >2M fragments within the defined size range. Of these different enzyme combinations, only *Bfa*I generated an attractive number of fragments (1.4M) with what seemed to be an approximately uniform size distribution of fragments (Figure 1a).

**Figure 1:**
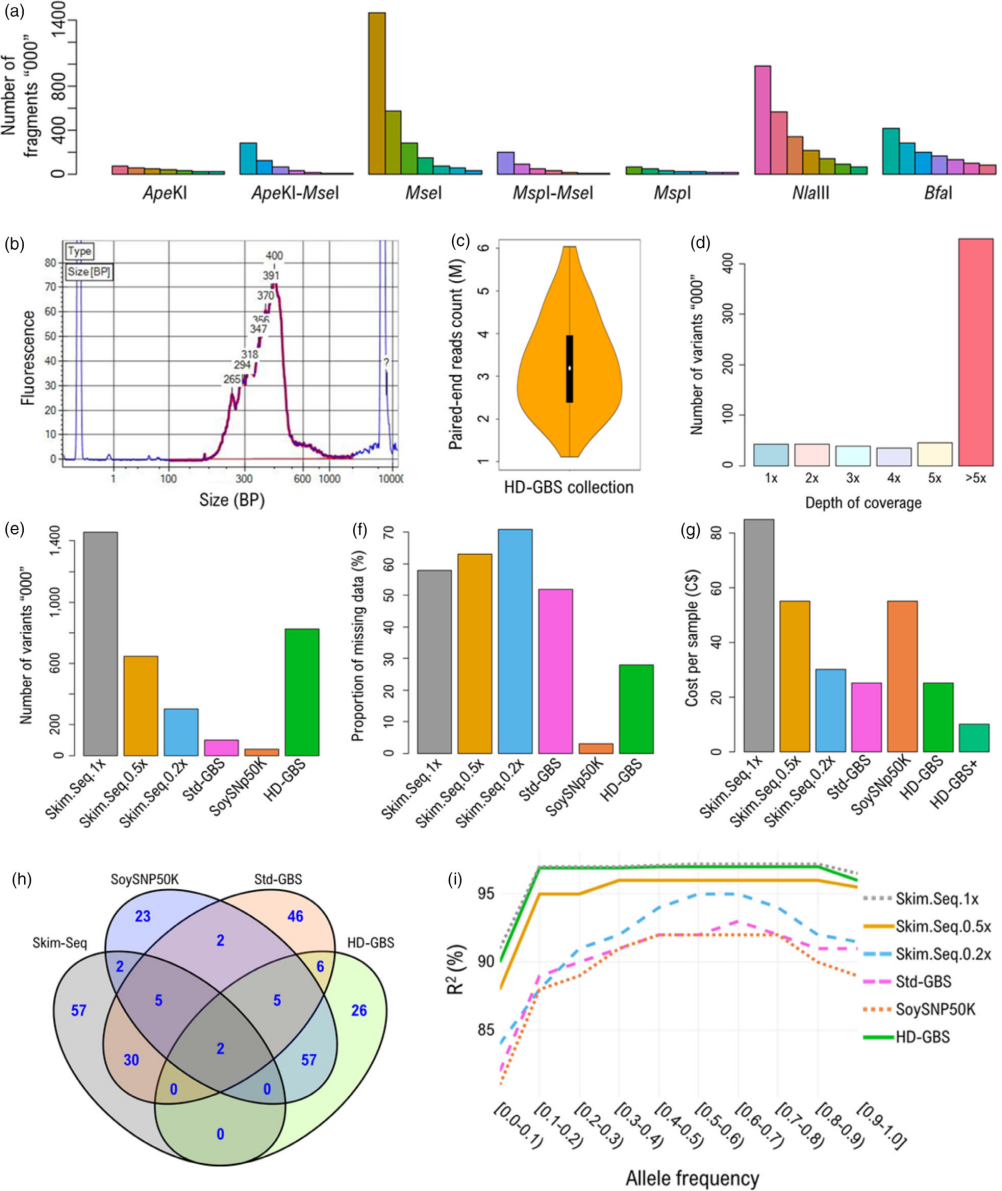
(a) Predicted distribution of fragments (100–800 bp; 100 bp bin size) derived from *in silico* digestion with seven different restriction enzymes or combinations thereof. (b) Quality assessment of a 96‐plex HD‐GBS library using a Bioanalyzer. (c) Distribution of PE reads per sample after demultiplexing. (d) Distribution of variants as a function of their depth of coverage following HD‐GBS. (e) Number of variants, (f) proportion of missing data and (g) cost per sample for six different genotyping platforms in soybean. HD‐GBS + is in the context of using NanoGBS protocol for library preparation and sequencing of 1500× multiplex with a single lane of Illumina NovaSeq 6000 (s4) platform. (h) Venn diagram representing the degree of overlap among samples used for genotyping using different genotyping platforms. (i) Accuracy of imputation as a function of allele frequency for six different genotypic datasets.

To validate these simulations experimentally, we constructed GBS libraries for a set of 96 diverse soybean accessions using *Bfa*I digestion and the standard GBS (Std‐GBS) protocol (Torkamaneh *et al*., 2020a). After size selection and PCR amplification, the quality of the GBS library was assessed and the resulting profile (Figure 1b) indicated that the vast majority of size‐selected fragments (including sequencing adapters) ranged between 200 and 600 bp. This reflects the effect of the size‐selection step which was chosen to favour fragments in the 200–600 bp window. Additionally, the profile was found to exhibit no primer dimers, no strong narrow spikes indicative of highly repetitive sequences nor PCR over‐cycling effect that can be detrimental. The DNA library had a concentration of 19 ng/μL that is suitable for DNA sequencing.

This GBS library was sequenced on a single lane of an Illumina HiSeq 4000 and generated 320M 150‐bp, paired‐end reads (publicly available at NCBI‐SRA, SRP262541). These reads were demultiplexed to yield an average of 3.3M PE reads per sample, on average (Figure 1c) and processed with the Fast‐GBS v2.0 (Torkamaneh *et al.,* 2020) pipeline for variant calling. Within this panel of 96 soybean accessions, 875 418 variants were obtained with 69% of variants supported by >5 reads (Figure 1d). This catalogue of variants contained only 28% missing data, and the SNPs were found to be well distributed across the genome.

Very large SNP catalogues (>500K SNPs) are now more and more common for GWAS studies in many crop species but, typically, this can only be achieved via WGRS and this is usually relatively costly compared with lower‐coverage approaches such as SNP arrays (SoySNP50K, 50K) or GBS (up to 200K) in soybean. Although affordable, the latter genotyping technologies do not yield a sufficient number of SNPs to ensure the capture of all haplotypes across the entire genome. Extremely low‐depth sequencing (<1×) such as Skim‐Seq (Happ *et al*., 2019) offers an interesting alternative to capture ~1M SNPs in soybean, but cost‐effective NGS library preparation and downstream data analysis (e.g. accurate missing data imputation) remain challenging. In contrast, HD‐GBS illustrates the potential of creating ultra‐dense catalogues of SNPs through GBS, an established genotyping approach offered by several service providers.

To assess the performance of HD‐GBS in terms of the number of variants, proportion of missing data and cost per sample, six genotypic data sets were created for 96 accessions including Skim‐Seq@1x (1.5M SNPs), Skim-Seq@0.5x (645K), Skim-Seq@0.2x (301K), Std‐GBS (98K), SoySNP50K (41K) and HD‐GBS (823K) (Figure 1e). The Skim‐Seq data sets were generated for the 96 samples by randomly sampling raw reads from the original WGRS data from the GmHapMap dataset (Torkamaneh *et al*., 2020b; 1007 accessions, 15M SNPs). SoySNP50K and Std‐GBS data for 96 samples were obtained from SoyBase and Torkamaneh *et al*. (2020b). The dramatically reduced depth of coverage of Skim‐Seq results in reduced cost per sample but this occurs at the expense of a higher proportion of missing data (>50%) (Figure 1f,g). HD‐GBS provides a lower‐cost method (25 vs 85 C$) for obtaining ultra‐dense genotypic information (823K) with a very low proportion of missing data (28%). Genotyping cost per sample can be dramatically decreased by miniaturizing library preparation (e.g. NanoGBS) and an increased multiplexing level (Torkamaneh *et al*., 2020a). We also developed a plate barcoding strategy (Colston‐Nepali *et al*., 2019) that enables the multiplexing of over 4500 GBS samples compatible with the highest throughput Illumina sequencing platforms (such as NovaSeq), thus enabling to tap into the economies of scale in sequencing (Figure 1g; HD‐GBS+).

All low‐cost approaches will require the imputation of missing genotypes and/or untyped variants from an extensively genotyped reference panel. The quality of imputation is highly dependent on the number variants in the lower‐density genotypic dataset (Browning *et al*., 2018). To assess the quality of imputation of untyped variants in soybean using the different low‐cost genotyping approaches, we performed imputation on different data sets. We used the GmHapMap dataset as a reference panel for imputation. The accuracy of imputation (squared correlation (R^2^) between imputed and known genotypes) ranged between 88% and 97% for common variants (allele frequency (AF) >0.1), with Skim‐Seq@1x and HD‐GBS achieving the same and highest degree of accuracy (97%) for allele frequencies between 0.1 and 0.8 (Figure 1h,i). Within this same window of allele frequencies, the genotyping approaches generating fewer than 100K SNPs (Std‐GBS and the SoySNP array) resulted in the lowest imputation accuracies, ranging between 88 and 90%. Finally, the other Skim‐Seq datasets (@0.5x and 0.2x), as expected, yielded accuracies falling between the two former categories. For the most extreme allele frequencies (<0.1 or >0.8), accuracies declined in a parallel fashion, based on the number of SNPs captured in the original catalogues. Imputing the rarest alleles (nearing an AF of 0) proved challenging as the accuracies for these more difficult cases ranged between 80 and 90%. Nonetheless, across all allele frequencies, HD‐GBS provided an imputation accuracy that was essentially identical to that achieved using the Skim‐Seq approach at the highest of the three depths of coverage tested (1×).

In conclusion, our results demonstrate that HD‐GBS provides an extremely low‐cost method for obtaining an ultra‐dense panel of markers enabling high‐quality imputation of untyped variants from a reference panel.

## Conflicts of interest

All authors declare no conflict of interest.

## Author contributions

DT and FB conceived the project and contributed to writing the manuscript. DLH provided DNA samples. DT and BB prepared DNA libraries and performed sequencing. DT and JL carried out data analysis.
